# The effect of smallholder maize farmers’ perceptions of climate change on their adaptation strategies: the case of two agro-ecological zones in Ghana

**DOI:** 10.1016/j.heliyon.2021.e08307

**Published:** 2021-11-01

**Authors:** Danley Colecraft Aidoo, Seth Dankyi Boateng, Comfort Kudadjie Freeman, Jonathan Nicholas Anaglo

**Affiliations:** Department of Agricultural Extension, University of Ghana, P. O. Box LG 68, Legon, Accra, Ghana

**Keywords:** Climate change adaptation, Adaptation strategies, Perceptions, Smallholder farmers, Agro-ecological zones

## Abstract

Maize is one of the most common cereals and a major staple that is cultivated across all agro-ecological zones in Ghana. However, maize productivity is affected by changes in climate, such as increased temperature and variations in rainfall. These changes in climate require farmers to implement practices (adaptation strategies) in order to reduce the magnitude of crop losses. This study examined how the perceptions of maize farmers regarding climate change affect their choice of adaptation strategies. A mixed methods approach was adopted for the study. Data was collected by means of a survey of 386 maize farmers along with focus group discussions. Quantitative data were analysed with descriptive statistics, Principal Component Analysis (PCA) and multivariate probit regression, while qualitative responses were used to explain the findings. Results from the data analysis indicated that maize farmers employed 17 adaptation strategies in adapting to climate change. The most common strategies identified were change of planting days, crop diversification, use of resistant varieties, and monitoring weather forecasts on radio. Furthermore, the factors that influenced the choice of adaptation strategies by maize farmers in both zones were experience in farming, household size, and perceptions about the impact and intensity of climate change.

## 1 Introduction

Maize is the most cultivated cereal crop in Ghana, accounting for up to half of the country's total cereal crop production (MOFA, 2017). In addition, it is one of the major staple crops produced by smallholder farmers in all agro-ecological zones in Ghana and is an essential component of animal feed (Mwambo et al., 2020; Scheiterle and Birner, 2016). Despite the importance of maize in the agricultural sector, changes in the climate pose some challenges in production. For example, due to erratic rainfall, farmers face the risk of total crop failure. Alternatively, inadequate amounts of rainfall at some stages of the growing season adversely affects crop growth and ultimately reduces total crop yield (MacCarthy et al., 2017). In adapting to the effects of climate change, rural farmers as well as formal research institutions have generated a number of adaptation strategies (Abdoulaye et al., 2017; Kuwornu et al., 2013). However, many smallholder farmers are not practicing the adaptation strategies (Fosu-Mensah et al., 2012). A major reason for this phenomenon is the perception smallholder maize farmers have about climate change. In order to adapt to climate change, farmers need to perceive ongoing changes as serious enough to warrant adaptation to the observed changes (Abid et al., 2015).

In their efforts to understand changes in climate, smallholder farmers give meaning to their experiences. In this regard, various studies have established several perceptions of smallholder farmers about the causes of climate change. For example, Moghariya and Smardon (2014) in their research conducted in Western India discovered that even though respondents perceived a change in climate due to their personal experiences they had no idea about the scientific context of climate change. Other studies reveal that in instances where smallholder farmers have identified changes in the climate, they have attributed these changes to non-scientific causes such as religious and moral factors (Tesfahunegn et al., 2016; Weber, 2010). Furthermore, farmers tend to perceive the severity of climate change indicators differently. While some farmers perceived increased rainfall, temperature, and flooding, other farmers perceived that these indicators were rather decreasing (Abdoulaye et al., 2017; Tesfahunegn et al., 2016).

The importance of these perceptions is borne in the fact that the perceptions of farmers concerning indicators of climate change significantly differ when compared across agro-ecological zones (Akponikpè et al., 2010; Mkonda et al., 2018). For instance, Kalungu and Harris (2013) observed that while 86% of farmers in the arid zone perceived low rainfall, only 38% of farmers in the sub-humid zone had a similar perception. Moreover, these perceptions differ even among farmers within an agro-ecological zone (Kalungu and Harris, 2013; Ndamani and Watanabe, 2016). In studies conducted in the mid-highland agro-ecological zone of Ethiopia, Tesfahunegn et al. (2016) observed that while farmers perceived increased rainfall and temperature, other farmers in the same zone perceived a decrease in rainfall and temperature. Thus, it is essential to consider the perceptions of farmers about climate change since it affects their adaptation to the effects of climate change (Ansari et al., 2018).

A greater portion of maize grown in Ghana is consumed locally making it an important crop for food security (Abdulai et al., 2017). Despite the importance of maize in the agricultural sector, changes in the climate pose some challenges in production. Due to erratic rainfall, farmers face the risk of total crop failure. Alternatively, inadequate amounts of rainfall at some stages of the growing season adversely affects crop growth and ultimately reduces total crop yield (MacCarthy et al., 2017).

In Ghana, various agricultural extension service providers have promoted several adaptation strategies. The main extension service providers are the Directorate of Agricultural Extension Services (DAES) of the Ministry of Food and Agriculture (MOFA), and non-governmental agencies (Asare-Nuamah and Botchway, 2019; Danso-Abbeam et al., 2018). The principal methods used by these institutions in providing agricultural extension services are the Training and Visit approach (T & V) and the transfer of technology approach to extension service delivery. The objective is to facilitate increased productivity by providing farmers with sufficient access to relevant information and technologies (Bonye et al., 2012). Despite the efforts of the various agricultural extension service providers to promote relevant adaptation strategies to farmers in Ghana, there exists a yield gap in maize production in Ghana. While the national average yield of farmers is 2.26 Mt/Ha, it is estimated that under rain fed conditions and the right management practices maize yields could potentially reach 5.5Mt/Ha (MOFA, 2019). Additionally, the Statistics, Research and Information Directorate (SRID) of the Ministry of Food and Agriculture (MOFA, 2019) indicated that even though productivity of maize marginally increased in 2018, the level of productivity achieved was 41.09% of the potential productivity.

Consequently, there is an urgent need to examine the various perceptions of farmers towards climate variability in order to understand the reasons underlying the choice of adaptation strategies. The study sought to investigate how smallholder farmers’ perceptions of climate change affect their choice of adaptation strategies.

## 2 Materials and methods

**Figure 1:**
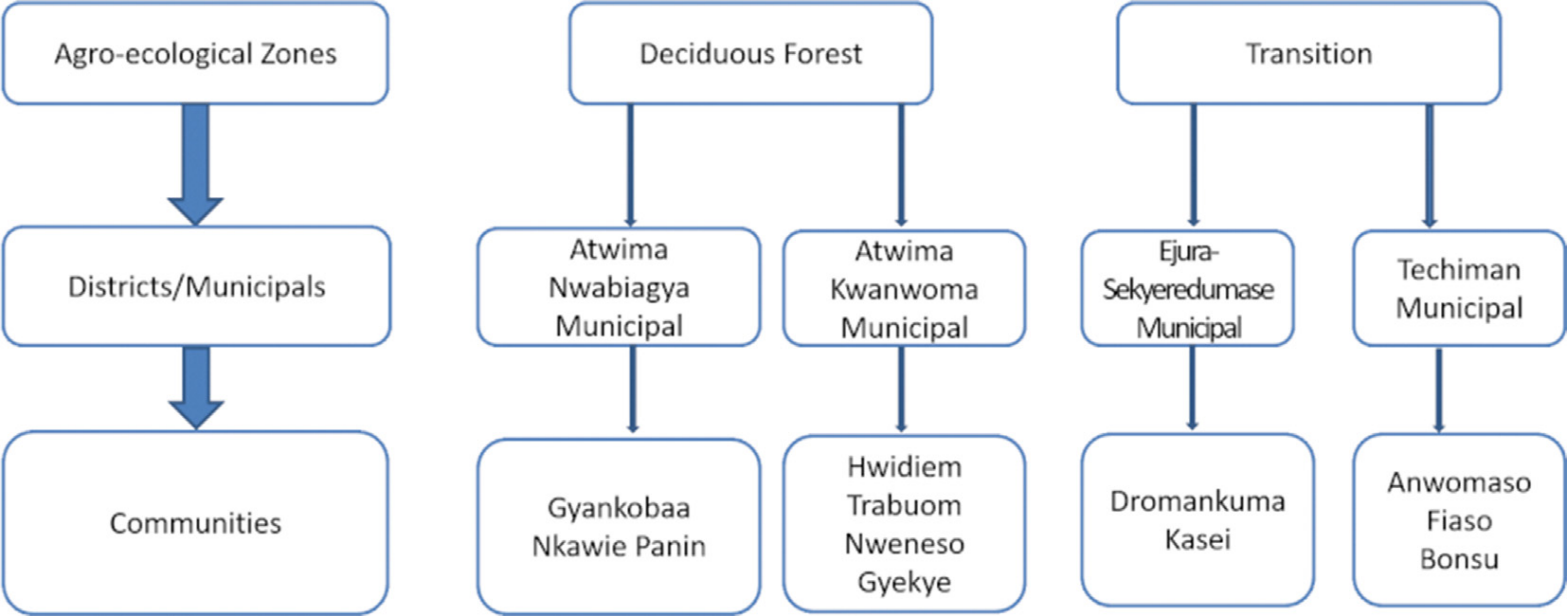
A framework for site selection.

Source: Authors' construct, 2020

The protocols used in this study were approved by the research ethics committee of the College of Basic and Applied Sciences, University of Ghana, Legon. The population for this study consisted of all smallholder maize farmers in Ghana. The various study areas were selected mainly based on smallholder farmers’ levels of maize production. First, the two highest maize producing agro-ecological zones, Deciduous Forest and Transitional zones were automatically selected (MOFA, 2019). Two districts and two municipals were chosen based on their significant role in maize production, budgetary limitations, and time in that order. Atwima Kwanwoma and Atwima Nwabiagya districts were selected from the Deciduous Forest agro-ecological zone (Figure 1), while the Ejura and Techiman Municipals were selected in the Transition agro-ecological zone. At this stage, communities that were primarily engaged in maize production and had an appreciable presence of smallholder maize farmers were purposively selected. Consequently, in the Deciduous Forest agro-ecological zone Gyankobaa, and Nkawie Panin were selected from the Atwima Nwabiagya municipal while Trabuom, Nweneso, Hwidiem, and Gyekye were selected from the Atwima Kwanwoma district. In the Transitional agro-ecological zone, Dromankuma and Kasei were selected from the Ejura Municipal while Aworowa, Bonsu, and Fiaso were chosen from the Techiman Municipal (see Figure 1).

All participants gave their informed consent orally. Face-to-face interviews were used to collect primary data from respondents using random sampling. Due to the lack of sufficient data on the entire population in the study area to design an accurate sampling frame, respondents were selected on the basis of farm size, gender, membership of Farmer Based Organizations (FBOs), accessibility, availability, and willingness to participate in the survey. In selecting respondents for focus group discussions, the snowball method of sampling was used owing to insufficient data on smallholder farmers in the study area. Consequently, community leaders were contacted during community entry and these served as points of referral for the first group of respondents. A major drawback of this method is the possibility of bias, considering the fact that the integrity of the sample is highly dependent on the first and consequent respondents (Abdul-Razak and Kruse, 2017). In order to minimize the effect of this bias, the researcher and enumerators sought numerous first respondents. In addition, efforts were made to select respondents from varied backgrounds and positions in the community. This was done by selecting multiple first responders on the basis of gender, farming experience, membership of FBOs, and participation in alternative sources of income.

Due to the lack of data regarding the total number of smallholder maize farmers in the selected agro-ecological zones, the sample size formula for an unknown population as proposed by Smith (2013) was adopted in determining the sample size. The formula is indicated in Eq. (1).

(1) $$\text{Sample ​size ​}=\frac{\left(\text{Z-score}\right)exp^{2}\times \text{ ​StdDev ​}\times \left(1-\text{StdDev}\right)}{\left(\text{margin ​of ​error}\right){\text{ ​exp}}^{2}}$$

where the Z-score at confidence level of 95% is 1.96, standard deviation is 0.5, and the margin of error (confidence interval) is 0.05

$$\text{Sample ​size ​}=\frac{\left(1.96\right)exp^{2}\times 0.5\times \left(1-0.5\right)}{\left(0.05\right){\text{ ​exp}}^{²}}=384\text{ ​respondents}$$

Accordingly, 384 smallholder maize farmers were sampled for the administration of questionnaires.

The primary data for the study was obtained from both quantitative and qualitative data sources. Quantitative data was acquired by administering pre-tested questionnaires while qualitative data was collected by means of a checklist during focus group discussions (FGDs). The questionnaires sought information on farmers’ perceptions of the causes of climate change and climate indicators, as well as their adaptation strategies. The focus group discussions were used to probe further into the perceptions of the respondents about climate change. Additionally, the focus group discussions elicited further information on strategies employed by smallholder farmers to improve their adaptation to climate change. There were ten focus group discussions, with five in each agro-ecological zone.

This study sought to evaluate how maize farmers' perceptions of climate change in the two agro-ecological zones affect their adaptation to climate change. The survey measured perceptions of smallholder farmers using questions regarding various climate variables (e.g. increase in rainfall, increase in temperature, onset of rainfall, strong winds, and flooding, etc.). The primary tool used for this study was a pretested interview schedule that was administered face-to-face. The perception questions assessing climate change patterns of the farmers had a 5-point Likert-scale responses ranging from *Strongly Disagree* to *Strongly Agree*. At the univariate level, the summary statistics of the perception statements were presented as percentages. By means of cross tabulations and Fisher's exact test of independence, indications of association between agro ecological zones and perception of climate change patterns was explored on the bivariate level. In addition, qualitative data obtained through focus group discussions were presented to confirm the quantitative results.

Adaptation was measured using 17 adaptation strategies identified from literature and focus group discussions conducted during pretesting. These were categorized into two namely, indigenous (12 strategies) and introduced (5 strategies) adaptation strategies. To identify the adaptation strategies employed by smallholder farmers to combat climate change, the 17 strategies were presented to the farmers during individual interviews. By means of descriptive statistics, their choices were analysed in SPSS. In determining the influence of perceptions of climate change on adaptation strategies, the independent variables in the analytical framework included eleven perception variables. These variables were run in a Principal Component Analysis (PCA) in order to shrink the high number of observed climate change perceptions to the principal variables observed as representative of an accurate measure of respondents’ climate change perceptions (Otieno et al., 2017). In addition, Principal Component Analysis (PCA) was used to assign weights to the various adaptation strategies in the two categories (introduced and indigenous strategies). These weights from the PCA computation provided the relative significance of each adaptation strategy in both categories and were used to calculate a total index per respondent for each category.

The Multivariate Probit regression model was used to estimate the relationship between smallholder farmers’ perceptions of climate change and their adaptation strategies. Farmers are more inclined to implement a combination of adaptation strategies to contend with various climate risks as compared to adopting a single adaptation strategy (Adimassu and Kessler, 2016; Bedeke et al., 2019). Accordingly, the decision of a farmer in climate change adaptation can be described as an essentially multivariate, mutually dependent, and concurrent decision (Lin et al., 2005; Tesfaye and Seifu, 2016). Thus, the multivariate probit regression model was used to estimate both observed and unobserved influence of the explanatory variables on major adaptation strategies identified. The variables used in the multivariate probit regression model were operationalized and defined as shown in Table 1.

**Table 1:** Description of variables used in the multivariate probit regression.

Variable	Description	Measurement
**Dependent Variable**		
*Adaptation Strategies*		
Shifting planting dates	If farmer shifted planting date	Dummy (1 yes, 0 no)
Move to different site	If farmer moved from one site to another	Dummy (1 yes, 0 no)
Change timing	If farmer changed timing of farm operations	Dummy (1 yes, 0 no)
Engage in extra income	If farmer had extra sources of income	Dummy (1 yes, 0 no)
Growing resistant variety	If farmer grew resistant maize variety	Dummy (1 yes, 0 no)
Use of weather forecast	If farmer used weather forecast information	Dummy (1 yes, 0 no)
Soil fertility management	If farmer employed soil fertility management methods	Dummy (1 yes, 0 no)
**Independent Variables**		
*Farmer Characteristics*		
Age	Age of the respondent	Age in years
Education	Level of education attained by respondent	1 = no formal education, 2 = basic education, 3 = secondary
Sex	Sex of respondent	1 = male, 2 = female
Household Size	Size of respondent's household	Count
Access to Credit	Access of farmer to credit	Dummy (1 yes, 0 no)
Access to Extension	Visits by extension agent in the past year	Dummy (1 yes, 0 no)
Labour	Total labour committed to the farm in man days	Days
Agro ecological zone	Agro ecological zone of the respondent	1 = Deciduous forest 0 = Transition
Experience	Number of years spent in maize farming	Years
Experience^2^	Number of years spent in maize farming squared	Years
*Perceptions*		
Timing	Perceptions about change in rainfall timing	Factor score
Amount	Perception about volume of rainfall	Factor score
Intensity	Perception about intensity of climate change	Factor score
Impact	Perception about effects of climate change	Factor score

Source: Authors' construct (2019)

## 3 Results

This section describes some socio-economic characteristics of the respondents. The characteristics presented are age, sex, marital status, household size, level of education, experience in maize farming, agro-ecological zones, and respondents’ primary source of income. Quantitative data was analysed by means of frequencies and percentages.

From the results in Table 2, most respondents (77.7%) were aged between 16 and 55 years, most common level of education reached among respondents was Junior High School (JHS) or Middle school whereas about 23.3% had not received any formal education. The remaining had been educated to Primary school (22.5), SHS/O/A Level (8.3%), and Vocational or Technical school level (1.6%).

**Table 2:** Socio-economic characteristics of respondents.

Variable	Categories	Frequency	Percentage
Sex	Male	248	64.2
	Female	138	35.8
	**Total**	**386**	**100**
Age	16–35	111	28.8
	36–55	189	48.9
	56–75	83	21.5
	76–95	3	0.8
	**Total**	**386**	**100**
Household Size	1–11	360	93.3
	12–21	25	6.4
	22–31	1	0.3
	**Total**	**386**	**100**
Level of Education	None	90	23.3
	Primary	87	22.5
	JHS/Middle School	171	44.3
	SHS/O/A Level	32	8.3
	Vocational/Technical	6	1.6
	**Total**	**386**	**100**
Experience	1–10	152	39.4
	11–20	104	26.9
	21–30	82	21.3
	31–40	31	8
	41–50	14	3.6
	51–60	3	0.8
	**Total**	**386**	**100**
Agro-Ecological Zone Of Respondents	Deciduous Forest	191	49.5
	Transition	195	50.5
	**Total**	**386**	**100**
Primary Source Of Income	Maize	231	59.8
	Alternative Sources	155	40.2
	**Total**	**386**	**100**

Source: Field Survey (2019)

The primary source of income for most farmers was maize (59.8%) while the remaining farmers (40.2%) had other primary sources of income such as growing other crops, sale of foodstuff, trading, construction, teaching, etc. In addition, 191 farmers (49.5%) were from the deciduous forest agro-ecological zone while 195 farmers (50.5%) were from the transition agro-ecological zone.

### 3.1 Smallholder farmers’ perception of climate change

The results presented in this section reveal maize farmers’ perception of climate change. The perceptions are presented and discussed in the following order: rainfall patterns, weather risks, and perceptions of climate change causes. The main type of data analysed was quantitative data and the findings were supported by qualitative data.

The findings indicate that majority of smallholder farmers in the transition zone (84.1%) identified increased rainfall as a significant evidence of climate change, while 49.2% from the deciduous forest zone with the same perception. In addition, while 52.9% of farmers in the deciduous forest zone did not perceive any decrease in rainfall amounts, a significantly higher number of farmers in the transition zone (85.1%) had the same perception.

Majority of farmers in the deciduous forest (53.9%) and transition (63.5%) zones also perceived that the rains occurred later than expected. This was corroborated by the perception of majority in both agro-ecological zones that early onset of rains was not a regular occurrence. In the deciduous forest zone, a majority of smallholder farmers (55%) perceived that the rainy season ends earlier than expected. On the other hand, a significantly low number of farmers in the transition zone (39%) agreed with this perception.

However, majority of farmers in both agro-ecological zones disagreed that there was a poor distribution of rainfall annually. As many as 85.2% in the transitional agro-ecological zone disagreed with this perception, while 67% in the deciduous forest zone believed that there was enough rainfall annually (Table 3).

**Table 3:** Smallholder maize farmers’ perceptions of rainfall patterns.

Rainfall pattern	Perception	Agro-ecological zone		Total	Fisher exact (P-Value)
		Deciduous forest	Transition		
Increased Rainfall	Agree	94 (49.2%)	164 (84.1%)	258 (66.8%)	0.00
	Neutral	25 (13.1%)	8 (4.1%)	33 (8.6%)	
	Disagree	72 (37.7%)	23 (11.8%)	95 (24.6%)	
	**Total**	**191 (100%)**	**195 (100%)**	**386 (100%)**	
Decreased Rainfall	Agree	76 (39.8%)	22 (11.2%)	98 (25.4%)	0.00
	Neutral	14 (7.3%)	7 (3.6%)	21 (5.4%)	
	Disagree	101 (52.9%)	166 (85.1%)	267 (69.2%)	
	**Total**	**191 (100%)**	**195 (100%)**	**386 (100%)**	
Late Onset Of Rains	Agree	103 (53.9%)	142 (63.5%)	245 (63.4%)	0.00
	Neutral	24 (12.6%)	11 (5.6%)	35 (9.1%)	
	Disagree	64 (33.5%)	42 (21.6%)	106 (27.5%)	
	**Total**	**191 (100%)**	**195 (100%)**	**386 (100%)**	
Early End Of Rains	Agree	105 (55%)	76 (39%)	181 (46.9%)	0.00
	Neutral	20 (10.5%)	33 (16.9%)	53 (13.7%)	
	Disagree	66 (34.5)	86 (44.1%)	152 (39.4%)	
	**Total**	**191 (100%)**	**195 (100%)**	**386 (100%)**	
Poor Distribution Of Rainfall	Agree	46 (24.1%	29 (14.9%)	75 (19.4%)	0.00
	Neutral	17 (8.9%)	0 (0%)	17 (4.4%)	
	Disagree	126 (67%)	166 (85.2%)	294 (76.2%)	
	**Total**	**191 (100%)**	**195 (100%)**	**386 (100%)**	

Source: Field Survey (2019)

The following narratives are representative of the general views about the onset of rain as expressed by maize farmers interviewed:

...*Looking back at when I used to farm with my grandfather, we used to expect the rains in early February so that by March we could begin planting our maize seeds. However, in recent times, we have to wait until as late as mid-June to plant our maize crops. That also seriously affects our yields for the minor season since we are unable to plant early enough after harvesting from the major season*. (A maize farmer in Hwidiem [deciduous forest agro-ecological zone], 16th June, 2019)

*...Previously, we had rains in January, right after celebrating Christmas. That helped us to prepare our fields to plant as early as February so that by July we would have harvested our maize and started to prepare for the minor season. Sadly, the rain now delays until as late as the month of April. This affects our yields and delays our planting activities for the minor season. This for me is the biggest evidence that our climate has changed.* (A maize farmer in Kasei [transition agro-ecological zone], 6th July, 2019)

*...we used to expect the rains early in the year so that we can plant our maize early. The rains usually extended until late March or mid-April, and the winds before those rains broke down some matured maize crops so that we usually had some ‘Good Friday maize’ during the Easter holidays to roast in our homes. For the past seven years, this has changed. The Easter holidays arrive and many of us would not have even planted our maize.* (A maize farmer in Bonsu [transition agro-ecological zone], 19th July, 2019)

In addition, the following statements describe the views commonly expressed by maize farmers interviewed about the end of the rains: *“The change in the duration of rains is very worrying. Just when the maize plants begin to tassel the rains end and it affects our yield a lot.”* (A farmer in Hwidiem [deciduous forest zone], 16th June 2019) *“Previously, once the rains started, we were assured of rains for at least two months. However, it has all changed now. We experience heavy rainfall that lasts for at most one month.”* (A farmer in Dromankuma [deciduous forest zone], 3rd July 2019).

Statistically significant differences in perceptions about rainfall patterns were observed between farmers in the two agro-ecological zones (p = 0.000). A possible reason for this observation is the possibility of rainfall variation within an agro-ecological zone. Consequently, smallholder farmers in more vulnerable areas of an agro-ecological zone had stronger perceptions of rainfall than those in less vulnerable areas. This is similar to the findings of Kangalawe (2017) and Mkonda et al. (2018) who observed that while rainfall patterns may be similar across different agro-ecological zones some zones experience severe variations than others in terms of regularity, intensity and levels of vulnerability.

These results reveal that smallholder maize farmers in both agro-ecological zone have similar perceptions of rainfall (increased amount, early onset of rains, and early end of rains) based on the existing climatic conditions in their locations. The results also showed that there were Considerable differences in the responses among the Farmers from different zones. Those from the most Vulnerable zones had stronger assertions than their Counterparts. They proclaimed that rainfall has signifi-cantly decreased in the are.

Respondents were also interviewed regarding their perceptions of weather risks such as flooding, increased temperatures, and occurrence of strong winds. In addition, smallholder farmers were interviewed concerning their perceptions of the intensity of climate change. The results show that almost all the respondents perceived a change in the climate except one respondent in the deciduous forest agro-ecological zone. However, there were differences in the perception of how intense these changes were. In the deciduous forest zone, 72.8% of respondents perceived the changes to be very intense, while 26.7% were of the view that the changes in climate was intense. On the other hand, 79.5% of respondents in the transition zone perceived very intense changes in the climate while 20.5% perceived the changes to be intense (Table 4).

**Table 4:** Smallholder maize farmers’ perceptions of weather risks.

Weather risk	Perception	Agro-ecological zone		Total	Fisher Exact (P-value)
		Deciduous forest	Transition		
Flooding	Agree	40 (20.9%)	48 (24.6%)	88 (22.8%)	0.12
	Neutral	14 (7.3%)	7 (8.7%)	31 (8.0%)	
	Disagree	137 (71.8%)	130 (66.7%)	267 (69.2%)	
	**Total**	**191 (100%)**	**195 (100%)**	**386 (100%)**	
Decreased Temperature	Agree	33 (17.3%)	35 (18%)	68 (17.6%)	0.93
	Neutral	10 (5.2%)	7 (3.6%)	17 (4.4%)	
	Disagree	148 (77.5%)	153 (78.4%)	301 (78.0%)	
	**Total**	**191 (100%)**	**195 (100%)**	**386 (100%)**	
Strong Winds	Agree	21 (11%)	31 (15.9%)	52 (13.5%)	1.68
	Neutral	17 (8.9%)	12 (6.2%)	29 (7.5%)	
	Disagree	153 (80%)	152 (78%)	305 (79.0%)	
	**Total**	**191 (100%)**	**195 (100%)**	**386 (100%)**	
Climate Change Intensity	Very Intense	139 (72.8%)	155 (79.5%)	294 (76.1%)	1.36
	Intense	51 (26.7%)	40 (20.5%)	91 (23.6%)	
	No Change	1 (0.5%)	0 (0%)	1 (0.3%)	
	**Total**	**191 (100%)**	**195 (100%)**	**386 (100%)**	

Source: Field Survey (2019)

The differences in perceptions among smallholder farmers in both zones with respect to flooding (p = 0.12), increased temperature (p = 0.930), strong winds (p = 1.68), and intensity of climate change (p = 1.36) were not statistically significant. Other studies in Ghana revealed similar trends. For example, in the Adansi North District in the deciduous forest zone, Asare-Nuamah and Botchway (2019) observed that most smallholder farmers perceived increased intensity of temperature, as well as late onset and early end of rainfall. Similarly, Yamba et al. (2019) in their study of the Bosomtwe district in the deciduous forest zone discovered that smallholder rural farmers experienced increased temperature, erratic rainfall patterns and flooding.

The differences in perceptions of climatic conditions within the agro-ecological zones could be attributed to variations in weather conditions prevalent in each community or district. Nyatuame et al. (2014) explain that these variations in weather conditions across communities or districts in an agro-ecological zone occur based on the presence or absence of mountains, thick forests, and water bodies such as rivers or lakes.

The study sought smallholder farmers’ perception of the causes of climate change by the use of open-ended questions. The responses were categorized into four namely, supernatural reasons, human activities, no knowledge and other factors (Table 5). In the deciduous forest zone 51.8% of respondents attributed the causes of climate change to supernatural reasons while 26.7% of farmers in the transition zone had a similar belief. However, the majority of farmers in the transition zone (50.8%) believed that climate change was due to human activities. This perception was also held by 32.5% of farmers in the deciduous forest zone. In addition, a few farmers indicated political factors as being responsible for climate change. In the transition zone, 1% had this perception while 1.6% had a similar perception in the deciduous forest agro-ecological zone.

**Table 5:** Smallholder maize farmers’ perceptions of climate change causes.

Agro-ecological zone	Deciduous	Transition	Total
Supernatural Reasons	99 (51.8%)	52 (26.7%)	151 (39.1%)
Human Activities	62 (32.5%)	99 (50.8)	161 (41.7)
No Knowledge	27 (14.1%)	42 (21.5%)	69 (17.9%)
Political Factors	3 (1.6%)	2 (1.0%)	5 (1.3%)
Total	191 (100%)	195 (100%)	386 (100%)
Fisher Exact (P-Value) p = 0.000			

Source: Field Survey (2019)

The results indicate that the difference between perceptions among smallholder maize farmers in the two agro-ecological zones is statistically significant (p = 0.00). From the results, majority of smallholder farmers in the deciduous forest zone (67.5%) either attributed the causes of climate change to non-scientific reasons or had no idea about what was responsible for the changes they had perceived in climatic conditions. In the transition zones however, a little over half of respondents (50.8%) attributed climate change to scientific factors.

The following statements are typical of the views of majority of the respondents who attributed climate change to supernatural reasons: *“...these changes are from the ancestors”*, *“...it is a sign from God that the end of the world is imminent”*, and *“...it is punishment from God because of the evil and wicked acts of people today.”*

Respondents in both agro-ecological zones commonly expressed the following responses during the focus group discussions: *“...the changes are caused by deforestation and bad farming activities such as burning of weeds, and excessive use of chemicals”* and *“...some of us are engaged in sand winning and illegal mining. As a result, the water bodies are drying up and affecting the climate.”*

These perceptions are similar to what Abdoulaye et al. (2017) discovered in the transition and savannah agro-ecological zones in Ghana. A possible reason for the seemingly low understanding of the causal factors of climate change could be due to the use of indigenous methods to forecast and estimate impending climatic situations. Previous studies have suggested that while such indigenous information allows local farmers to form their perceptions of climate change, they are usually inconsistent and may differ from person to person within a community (Ishaya and Abaje, 2008; Mengistu, 2011). Accordingly, indigenous and religious beliefs can have a bearing on the ability of smallholder farmers to modify their traditional and farming activities to enable them adapt to the effects of climate change. In view of the importance of religious and indigenous beliefs in smallholder famers’ understanding and perceptions of climate change, it is necessary for change agents to identify these factors when seeking to improve the attitudes and practices of indigenous farmers.

### 3.2 Adaptation strategies employed by smallholder farmers

This section presents the results of adaptation strategies employed by smallholder farmers. The perceptions are presented and discussed in the following order: indigenous adaptation strategies and introduced adaptation strategies. Quantitative data was primarily used in the analysis.

The study discovered 17 adaptation strategies employed by smallholder maize farmers in the two agro-ecological zones. Of this number, 12 were indigenous strategies generated by farmers themselves, while formal research institutions and extension services providers introduced the remaining strategies.

In the deciduous forest zone (see Table 6), a few farmers employed planting local maize varieties (8.4%), adding organic matter (23%), planting shade trees (17.8%), and change in timing of farm operations (29.3%). The most common adaptation strategies among farmers in the deciduous forest zone were shifting planting dates (61.3%), and crop diversification (52.9%).

**Table 6:** Indigenous strategies used by smallholder maize farmers.

Adaptation strategy	Choice of respondents	Agro-ecological zones		Total	Fisher's Exact (P-Value)
		Deciduous forest	Transition		
Planting Local Variety	Yes	16 (8.4%)	24 (12.3%)	40 (10.4%)	0.243
	No	175 (9.6%)	171 (87.7%)	346 (89.6%)	
	**Total**	**191**	**195**	**386 (100%)**	
Shifting Planting Dates	Yes	117 (61.3%)	126 (64.6%)	243 (63.0%)	0.528
	NO	74 (38.7%)	69 (35.4%)	143 (37.0%)	
	**Total**	**191**	**195**	**386 (100%)**	
Change To Different Crop	Yes	50 (26.2%)	20 (10.3%)	70 (18.1%)	0.000
	No	141 (73.8%)	175 (89.7%)	316 (81.9%)	
	**Total**	**191**	**195**	**386 (100%)**	
Move To A Different Site	Yes	64 (33.5%)	50 (25.6%)	114 (29.5%)	0.095
	No	127 (66.5%)	145 (74.4%)	272 (70.5%)	
	**Total**	**191**	**195**	**386 (100%)**	
Diversify Crops	Yes	101 (52.9%)	87 (44.6%)	188 (48.7%)	0.127
	No	90 (47.1%)	108 (55.4%)	198 (51.3%)	
	**Total**	**191**	**195**	**386 (100%)**	
Water Harvesting	Yes	6 (3.1%)	8 (4.1%)	14 (3.6%)	0.787
	No	185 (96.9%)	187 (95.9%)	372 (96.4%)	
	**Total**	**191**	**195**	**386 (100%)**	
Add Organic Matter	Yes	44 (23.0%)	4 (2.1%)	48 (12.4%)	0.000
	No	147 (77%)	191 (97.9%)	338 (87.6%)	
	**Total**	**191**	**195**	**386 (100%)**	
Planting Shade Trees	Yes	34 (17.8%)	21 (10.8%)	55 (14.2%)	0.058
	No	157 (82.2%)	174 (89.2%)	331 (85.8%)	
	**Total**	**191**	**195**	**386 (100%)**	
Change To Livestock Management	Yes	14 (7.3%)	1 (0.5%)	15 (3.9%)	0.000
	No	177 (92.7%)	194 (99.5%)	371 (96.1%)	
	**Total**	**191**	**195**	**386 (100%)**	
Mulching	Yes	19 (9.9%)	22 (11.3%)	41 (10.6%)	0.742
	No	172 (90.1%)	173 (88.7%)	345 (89.4%)	
	**Total**	**191**	**195**	**386 (100%)**	
Change Timing Of Farm Operations	Yes	56 (29.3%)	69 (35.4%)	125 (32.4%)	0.232
	No	135 (70.7%)	126 (64.6%)	261 (67.6%)	
	**Total**	**191**	**195**	**386 (100%)**	
Engage In Extra Income Sources	Yes	79 (41.4%)	67 (34.4%)	146 (37.8%)	1.73
	No	112 (58.6%)	128 (65.6%)	240 (62.2%)	
	**Total**	**191**	**195**	**386 (100%)**	

Field Survey (2019).

The p-values indicated that there were no significant statistical differences between farmers in both agro-ecological zones with respect to several indigenous adaptation strategies. This suggests that notwithstanding their agro-ecological zones, smallholder farmers took similar actions regarding their choice of strategy in adapting to perceived effects of climate change. An explanation for this phenomenon is the fact that smallholder farmers in both zones suffer similar climate variabilities, although on different scales of intensity. On the other hand, there were statistically significant differences among farmers in the agro-ecological zones with respect to changes in different crops (p = 0.000), addition of organic matter to soils (p = 0.00), and changing to livestock management. This implies that even though smallholder farmers applied particular adaptation strategies across both agro-ecological zones in order to adapt to climate change they had different purposes for doing so.

Respondents in both agro-ecological zones indicated five main strategies that had been introduced to them by the Department of Agricultural Extension of the Ministry of Food and Agriculture and other non-governmental organizations. The major adaptation strategy that was identified among farmers in the deciduous (70.7%) and transition (61.5%) was the use of weather forecast (Table 7). On the other hand, the least employed adaptation strategies were irrigation and agroforestry.

**Table 7:** Introduced strategies used by smallholder maize farmers.

Adaptation strategy	Choice of respondents	Agro-ecological zones		Total	Fisher's Exact (P-Value)
		Deciduous forest	Transition		
Growing Resistant Variety	Yes	92 (48.2%)	44 (22.6%)	136 (35.2%)	0.000
	No	99 (51.8%)	151 (77.4%)	250 (64.8%)	
	**Total**	**191**	**195**	**386 (100%)**	
Use Of Irrigation	Yes	3 (1.6%)	1 (0.5%)	4 (1.0%)	0.304
	No	188 (98.4%)	194 (99.5%)	382 (99.0%)	
	**Total**	**191**	**195**	**386 (100%)**	
Weather Forecast	Yes	135 (70.7%)	120 (61.5%)	255 (66.1%)	0.068
	No	56 (29.3%)	75 (38.5%)	131 (33.9%)	
	**Total**	**191**	**195**	**386 (100%)**	
Soil Fertility Management	Yes	76 (39.8%)	27 (13.8%)	103 (26.7%)	0.000
	No	115 (60.2%)	168 (86.2%)	283 (73.3%)	
	**Total**	**191**	**195**	**386 (100%)**	
Agroforestry	Yes	15 (7.9%)	0 (%)	15 (3.9%)	0.000
	No	176 (92.1%)	195 (100%)	371 (96.1%)	
	**Total**	**191**	**195**	**386 (100%)**	

Field Survey (2019).

The following statements typify the general views of farmers interviewed in connection with the use of weather forecast: In the deciduous zone, a farmer said:

*...even though it is not perfect, the weather forecast is very helpful. I listen to ‘Wofa Ankomah’ (radio presenter) on Fox FM every morning before I go to the farm. On several occasions, it has saved me from wasting my chemicals.* (A maize farmer in Trabuom [deciduous forest zone], 13th June 2019)

*“The weather forecasts from various radio stations such as Nana Adjei FM, Achiaa Radio, Peace FM and Adom FM help us to predict rainfall. Things are better than they were previously because we are well educated now. Previously I used to get about five bags but now I am able to get almost 10 bags from 1 acre of land. It has greatly improved my life.”* (A maize farmer in Dromankuma [transition zone], 3rd July 2019)

With respect to irrigation, very few farmers in both agro-ecological zones made use of the adaptation strategy. In the deciduous zone, just three farmers used irrigation while just one farmer in the transition zone used irrigation to combat insufficient rainfall. This finding is similar to other studies that found irrigation to be one of the poorly adopted strategies. Moreover, those who used irrigation applied traditional indigenous methods that did not significantly improve their resilience to inadequate rainfall (Mkonda et al., 2018; Sieber et al., 2015). Probably this phenomenon arises due to insufficient information and technology to allow smallholder farmers utilize potential irrigation sources such as groundwater and harvesting rainfall. Farmers in both agro-ecological zones mentioned challenges that made it difficult to practice irrigation. The general views of maize farmers interviewed are characterised by these statements: *“We have streams that we can fall on when the rainfall patterns change, but due to excess sunlight these waterbodies dry up easily so we are not allowed to use the water for our plants.”* (A farmer in Fiaso [transition zone], 20th July 2019)

*“The river is far from our farms, so if the rains fail us there is no other way to water our farms. The option of drawing water from the river to our farms is not viable due to the distance and cost involved.”* (A farmer in Hwidiem [deciduous forest zone], 16th June 2019)

### 3.3 Influence of climate change perceptions on adaptation strategies used by smallholder farmers

The results presented in this section reveal how maize farmers’ perception of climate change affect the adaptation strategies they implement. The data used in this analysis was solely quantitative. Qualitative data was used in the discussions to support some aspects of the findings.

Before conducting the multivariate probit regression, the principal component analysis (PCA) technique was employed as a data reduction method to confirm that the perception factors used were accurately measured and significantly accounted for the variability in the model. From the varimax-rotated PCA results as shown in Table 8, four factors were extracted based on the climate change perceptions of the respondents. The generated variables were (i) volume of rain, (ii) timing of rain, (iii) intensity of climate change, and (iv) impact of climate change. The independent variables generated from PCA were regressed against the dependable variable (indigenous and introduced adaptation strategies). The generated variables were used in the multivariate probit model.

**Table 8:** PCA results on respondents’ perceptions of climate change.

Rotated Component Matrix^a^				
	Component			
	1 = Volume of rain	2 = Timing of rain	3 = Intensity	4 = Impact
More rain	.918			
Less rain	.925			
Early onset of rain		.938		
Late onset of rain		.937		
Poor distribution of rainfall				.674
Frequent flooding				.577
High temperature			.739	
Strong winds			.719	

Author's computation (2019).

Extraction Method: Principal Component Analysis.

Rotation Method: Varimax with Kaiser Normalization.

a ∖Rotation converged in 5 iterations.

The multivariate probit model results as presented in Table 9 indicate that personal characteristics such as agro-ecological zone, sex, and experience of respondents affected the decision to adopt various adaptation strategies.

**Table 9:** Coefficient estimates of Multivariate Probit regression model results.

Dependent Variables	Indigenous Adaptation Strategies				Introduced Adaptation Strategies		
	Shifting Planting Dates	Move to a Different Site	Change Timing of Farm Operations	Engage in extra income sources	Growing resistant variety	Use of weather forecast	Soil fertility management
**Variables**	*Coefficient (Std. Error)*	*Coefficient (Std. Error)*	*Coefficient (Std. Error)*	*Coefficient (Std. Error)*	*Coefficient (Std. Error)*	*Coefficient (Std. Error)*	*Coefficient (Std. Error)*
Age	-.008 (.008)	.009 (.008)	-.004 (.010)	.008 (.010)	.005 (.007)	-.006 (.008)	-.005 (.008)
Sex	.170 (.153)	.147 (.176)	-.200 (.210)	.138 (.205)	.013 (.151)	.493∗ (.166)	.101 (.178)
Household Size	.015 (.022)	-.024 (.029)	.048 (.031)	-.007 (.039)	-.004 (.022)	.019 (.024)	-.019 (.030)
Experience	.032∗∗∗ (.019)	.091∗ (.027)	.023 (.032)	.016 (.034)	.028 (.020)	.047∗∗ (.020)	.019 (.023)
**Experience**^**2**^	-.000 (.000)	-.002∗ (.001)	-.001 (.001)	-.010 (.001)	-.001∗ (.000)	-.001 (.000)	-.001 (.001)
Extension Visit	-.294 (.200)	-.174 (.215)	.392 (.265)	-.792∗ (.296)	.620∗ (.192)	-.623∗ (.221)	.027 (.216)
Access to Credit	-1.129∗ (.368)	-.848 (.556)	.036 (.565)	-.354 (.630)	-.088 (.362)	-.941∗∗ (.420)	-.486 (.510)
Labour	.000 (.009)	.008 (.011)	-.020 (.017)	-.004 (.013)	-.008 (.009)	-.006 (.010)	.011 (.011)
Volume	-.326∗ (.082)	-.029 (.093)	.133 (.104)	.050 (.104)	.089 (.079)	-.199∗∗ (.091)	.073 (.091)
Impact	-.215∗ (.076)	.190∗∗ (.091)	.004 (.113)	.077 (.108)	-.074 (.076)	-.180∗∗ (.083)	.079 (.089)
Intensity	-.005 (.075)	-.421∗ (.089)	-.107 (.111)	-.329∗ (.106)	.023 (.076)	-.150∗∗∗ (.081)	-.421∗ (.093)
Timing	-.079 (.074)	.081 (.084)	.191∗∗ (.106)	.157 (.100)	.011 (.073)	.017 (.082)	.110∗ (.086)
Agro-ecological zone	.019 (.176)	.618∗ (.204)	1.500∗ (.321)	.596∗ (.231)	.646∗ (.172)	.369∗∗∗ (.193)	1.337∗ (.215)
Constant	-.952∗ (.395)	-2.820∗ (.494)	-2.941∗ (.617)	-3.015∗ (.580)	-1.782∗ (.408)	-2.221∗ (.449)	-2.564∗ (.487)

Note: ​∗, ∗∗ and ∗∗∗ indicate significance at 1%, 5% and 10%, respectively.

Model summary; No of observations 386; Wald chi-square (95%) 267.45; Log likelihood -566.44284.

Source: Field Survey (2019).

The results indicate that sex positively influences the use of introduced adaptation strategies. Specifically, male smallholder maize farmers were more likely to use information from weather forecasts. This is in agreement with studies such as Mulwa et al., (2017) that found male headed households more likely to make use of adaptation strategies due to their control of assets. In addition, farmers' experiences positively affected indigenous strategies such as shifting in planting dates and moving to different sites. Experience was also found to influence the implementation of both indigenous (shifting planting dates and moving to a different site) and introduced strategies (use of weather forecasts) positively. However, the results for experience squared point to a negative relationship with the choice of moving to a different site (indigenous strategy) as well as planting resistant varieties (introduced strategy). These outcomes reveal that farming experience is likely a motivating factor in a farmer's decision to respond to the harmful effects of climate change on maize production. This is likely because experienced farmers are likely to be more cognizant of previous climate events and may consequently decide to use adaptation strategies as a response to changes in climate. Additional support for this explanation is seen in studies by Diallo et al. (2020) and Sadiq et al. (2019) who also found that experience significantly influences the use of adaptation strategies.

Nevertheless, the negative results for experience squared mean that at some point, the effect of farming experience on adaptation strategies is lessened. This can be attributed to the fact that some of the adaptation strategies are either labour or cost intensive. Consequently, experienced farmers at some point may be unable to implement adaptation strategies since they may lack the required financial and physical abilities, in addition to labour. In line with this observation, some studies have discovered that younger farmers are more likely to use labour intensive strategies in adapting to the effects of climate change as compared to the elderly (Falola and Achem, 2017; Uddin et al., 2014). For example, Alhassan et al. (2018) in their study of the Guinea Savannah and Savannah agro-ecological zones of Ghana found that even though elderly farmers were experienced, they were unable to implement labour-intensive adaptation strategies.

In contrast with other findings (Abid et al., 2016; Ojo and Baiyegunhi, 2018), access to extension services had a negative influence on implementing indigenous (engaged in sources of extra income) and introduced (use of weather forecast information) strategies. On the other hand, farmers who had access to extension services were more likely to plant resistant varieties of maize in adapting to climate change. This highlights the significance of information provided by extension agents in combating climate change. Ojo and Baiyegunhi (2018) explain that farmers who have access to climate information as provided by extension agents are more likely to quickly adapt to risks related to climate change. Furthermore, the results show that access to credit has a significant influence on a smallholder maize farmer's possibility of using indigenous and improved strategies. Farmers without access to credit were more likely to implement strategies such as shifting planting dates and the use of weather forecast information. Due to financial constraints, smallholder farmers are usually unable to implement climate change adaptation strategies that involve financial costs and resources (Mulwa et al., 2017). Consequently, farmers without access to credit preferred adaptation strategies that were less resource and cost intensive.

The agro-ecological zone of farmers showed a positive and significant relationship with both indigenous and introduced adaptation strategies. Smallholder maize farmers in the deciduous forest agro-ecological zone had a higher possibility of implementing indigenous and adaptation strategies as compared to farmers in the transition zone. Similarly, previous research studies have shown that due to differences in climatic conditions, soil fertility and other factors, the location of farmers is a significant determinant in the choice and implementation of adaptation strategies (Ojo and Baiyegunhi, 2018; Tesfaye and Seifu, 2016).

### 3.4 Influence of perceptions on choice of adaptation strategies

The results from the multivariate probit regression indicate that the perception of climate change intensity had a negative and significant effect on the use of most indigenous and introduced strategies. Consequently, this implies that farmers who perceived intense climate change were less likely to implement both introduced and indigenous strategies. A possible explanation for this result is that farmers probably do not view the adaptation strategies as effective enough; consequently, they are reluctant to apply adaptation strategies or modify their practices.

Respondents expressed similar viewpoints during the focus group discussions. The statements indicated below are typical of the general view of respondents: *“The changes we are seeing are acts of God. What can we do about it? We just have to pray that God has mercy on us…”* In the transition agro-ecological zone, there were similar sentiments expressed by farmers. In Kasei, a farmer expressed the following opinion: *“…There is nothing we can do about the changes. We cannot force the rain to obey us and we cannot tell the sun when to shine. We just hope that things change for us in the future.”*

*No matter what we do, we cannot change what is happening. Maybe the government and the big men in the cities can help us with funds so that we will be able to fight against these changes. Otherwise, we can do little.* (A maize farmer in Dromankuma [transition zone], 3rd July 2019)

These results are consistent with the findings of Mitter et al. (2019) that some farmers who have a perception of an increased incidence of extreme climate change do not apply adaptation strategies because they view such strategies as ineffective or too costly for their farms.

In a similar vein, the perceptions that there were changes in the volume of rainfall and the impact of climate change displayed a negative, significant effect on the use of indigenous (shift in planting dates) and introduced strategies (use of weather forecast). This suggests that farmers who perceived a change in rain or severe impact of climate change were not likely to employ most adaptation strategies in their farming activities. However, farmers who did not perceive the increased impact of climate change were more likely to move to a different site (indigenous strategy).

Furthermore, the perception that there was a change in the timing of rainfall positively affected the use of indigenous (change of timing of farm operations) and introduced (soil fertility management) adaptation strategies. This suggests that farmers who perceived a change in timing of rainfall were more likely to implement adaptation strategies with the aim of combating climate change risks.

## 4 Conclusions

It is concluded in the study that maize farmers in the two agro-ecological zones have ample perceptions of climate change indicated in increased rainfall, late onset of rains, increased temperature due to intense sunshine, as well as erratic rainfall. The use of the multivariate probit regression model permitted the synchronized identification of the determinants of the adaptation options identified. The multivariate probit regression results revealed that farmers' perceptions of intensity and impact of climate change, access to credit, experience in maize farming, access to extension services, and the agro-ecological zone of farmers were major determinants of maize farmers' decision to implement both indigenous and introduced adaptation strategies. In addition, the study has highlighted the significant influence of farmers’ perceptions of climate change on their choice of adaptation strategies.

These findings highlight the importance of farmers' perceptions of climate change in extension delivery and farmer education. Extension agents should intensify their efforts in educating farmers in using introduced adaptation strategies such as the use of improved seeds, use of weather forecast information, irrigation, and agrochemicals in combating the effects of climate change. Climate change adaptation policies will need to take into account farmers' perceptions and knowledge of climate change causes, patterns, and effects. Considering that access to credit was a significant determinant of both indigenous and introduced adaptation strategies, it is necessary for stakeholders to create an enabling environment to increase smallholder farmers' access to credit facilities. This will contribute to enhancing smallholder farmers’ ability to implement resource intensive adaptation strategies.

As with most research studies, there are limitations that offer opportunities for further research. Considering that this was a cross-sectional study, it remains to be seen how smallholder farmers' perceptions of climate change evolve over time along with any significant changes in their influence on the choice of farmers’ adaptation strategies.

## Declarations

### Author contribution statement

Danley Colecraft Aidoo: Conceived and designed the experiments; Performed the experiments; Analyzed and interpreted the data; Wrote the paper.

Seth Dankyi Boateng; Comfort Kudadjie Freeman; Jonathan Nicholas Anaglo: Conceived and designed the experiments; Analyzed and interpreted the data.

### Funding statement

This work was supported by USAID-GHANA/10.13039/501100005601University of Ghana Institutional Capacity Building to Improve Agricultural Productivity and Food Security in the Context of Economic Policy Management in Ghana project.

### Data availability statement

Data will be made available on request.

### Declaration of interests statement

The authors declare no conflict of interest.

### Additional information

No additional information is available for this paper.
